# Extremophilic microbial communities on photovoltaic panel surfaces: a two‐year study

**DOI:** 10.1111/1751-7915.13620

**Published:** 2020-07-01

**Authors:** Kristie Tanner, Esther Molina‐Menor, Adriel Latorre‐Pérez, Àngela Vidal‐Verdú, Cristina Vilanova, Juli Peretó, Manuel Porcar

**Affiliations:** ^1^ Darwin Bioprospecting Excellence S.L. Calle Catedrático Agustín Escardino 9 Paterna 46980 Spain; ^2^ Institute for Integrative Systems Biology I2SysBio University of Valencia – CSIC Catedrático José Beltrán 2 Paterna 46980 Spain; ^3^ Department of Biochemistry and Molecular Biology University of Valencia Dr. Moliner 50 Burjassot 46100 Spain

## Abstract

Solar panel surfaces can be colonized by microorganisms adapted to desiccation, temperature fluctuations and solar radiation. Although the taxonomic and functional composition of these communities has been studied, the microbial colonization process remains unclear. In the present work, we have monitored this microbial colonization process during 24 months by performing weekly measurements of the photovoltaic efficiency, carrying out 16S rRNA gene high‐throughput sequencing, and studying the effect of antimicrobial compounds on the composition of the microbial biocenosis. This is the first time a long‐term study of the colonization process of solar panels has been performed, and our results reveal that species richness and biodiversity exhibit seasonal fluctuations and that there is a trend towards an increase or decrease of specialist (solar panel‐adapted) and generalist taxa, respectively. On the former, extremophilic bacterial genera *Deinococcus*, *Hymenobacter* and *Roseomonas* and fungal *Neocatenulostroma*, *Symmetrospora* and *Sporobolomyces* tended to dominate the biocenosis; whereas *Lactobacillus* sp or *Stemphyllium* exhibited a decreasing trend. This profile was deeply altered by washing the panels with chemical agents (Virkon), but this did not lead to an increase of the solar panels efficiency. Our results show that solar panels are extreme environments that force the selection of a particular microbial community.

## Graphical abstract

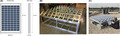

## Introduction

Extreme environments are characterized by their strong selective pressures, which can include physical (i.e., temperature or radiation), geochemical (i.e., desiccation or salinity) and/or biological stresses (i.e., limited nutrient availability) (Lynn and Rocco, 2001). The microorganisms that inhabit these environments, known as extremophiles or extremotolerants, are selected due a variety of mechanisms, such as biofilm formation (Flemming *et al*., 2016; Blanco *et al*., 2019); the production of extremolytes and extremozymes (Gabani and Singh, 2013); or highly efficient DNA repair systems (Singh and Gabani, 2011). Microorganisms inhabiting extreme environments evolve faster than those inhabiting ‘benign’ environments, mainly due to the high mutation rates associated to stressful environmental conditions (Li *et al*., 2014), and this could lead to these microorganisms being rich sources of new specialized metabolites (Sayed *et al*., 2019).

A diversity of physical, geochemical and biological extremes (solar radiation, temperature fluctuations, desiccation and limited nutrient availability) concur on solar panel surfaces. A study performed on subaerial solar panel biofilms in São Paulo revealed that dust, pollen and other debris covering the solar panel surfaces accumulated in time and included abundant fungi and pigmented bacterial genera, and this was associated with a decrease in the photovoltaic power efficiency, especially after 12 and 18 months (loss of 7% and 11% power respectively) (Shirakawa *et al*., 2015). This process – the accumulation of dust particles and microorganisms on a surface – is known as soiling, and it affects photovoltaic efficiency especially under dry and arid conditions, such as those in the Atacama Desert, resulting in an annual energy loss of up 39% in regions with infrequent rainfalls (Cordero *et al*., 2018).

Microbial colonization of solar panel surfaces is of great interest not only from an energetic point of view, but also from an ecological perspective. The widespread distribution around the world of these artificial devices, as well as their relatively standard design, has enabled them to be used as ubiquitous sampling devices for microbial ecologists in the recent years. A previous study of solar panels located in Valencia (Spain) revealed that these surfaces are inhabited by diverse, desert‐like microbial communities that show different day/night proteomic profiles and are adapted to high temperatures, desiccation and solar radiation (Dorado‐Morales *et al*., 2016). The microbial communities present on the solar panels from Valencia proved rather similar, in taxonomic terms, to those on solar panels located in Arctic and Antarctic regions, with the most abundant genera being *Hymenobacter*, *Sphingomonas* and *Deinococcus* in all cases (Tanner *et al*., 2018). Furthermore, the microbiome of solar panel surfaces from Berkeley (California, USA) also displayed similar profiles, both in taxonomic and functional terms, to those observed on the Spanish solar panels, highlighting the role of selective pressures in the establishment of these microbial communities (Porcar *et al*., 2018). Nevertheless, and despite the previous taxonomic and functional characterization of the solar panel microbiome, little is known about the colonization process of these surfaces.

In the present study, we have weekly monitored the photovoltaic efficiency of 54 small‐sized solar panels, and we have analysed the microbiome composition – including fungi and bacteria – every seven weeks, throughout a period of two years, with the aim of studying in detail the microbial colonization process and its effect on photovoltaic efficiency. Furthermore, we have assessed the effect on the solar panel microbiome of periodically treating the solar panel surfaces with a disinfectant.

## Results

**Fig. 1:**
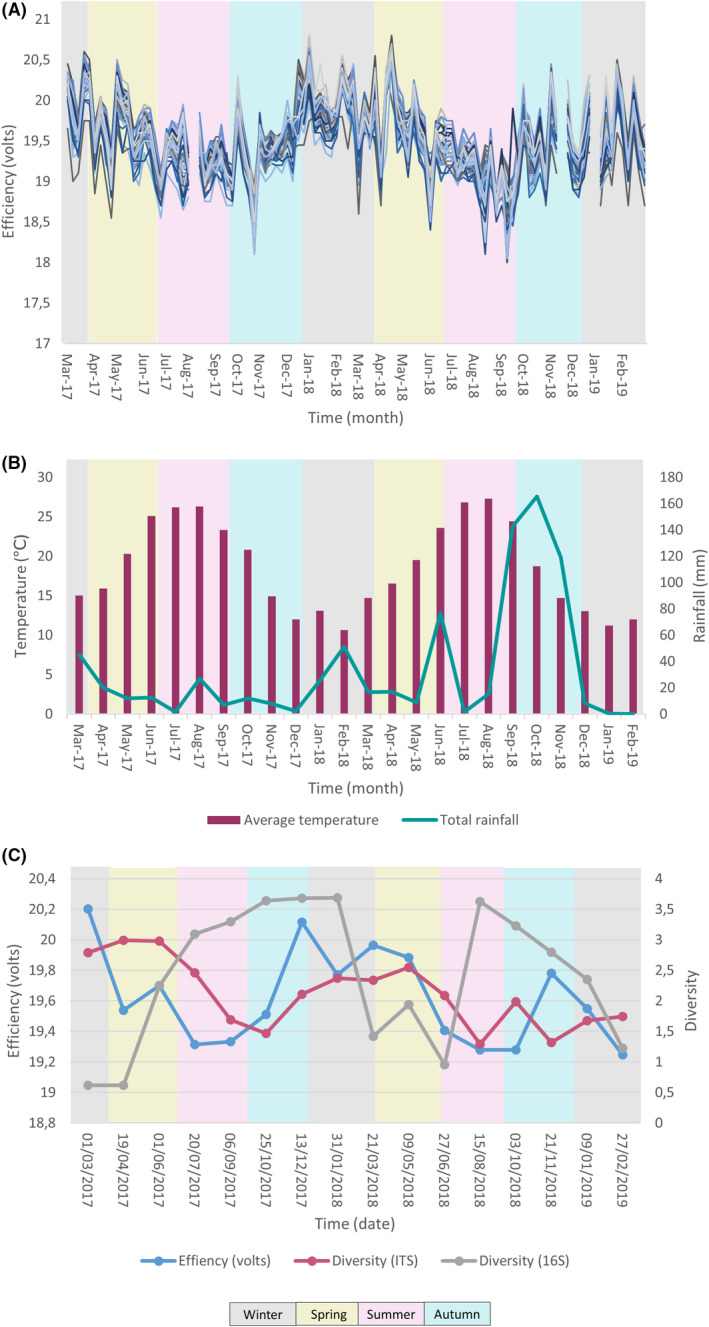
(A) Variations in solar panel voltage throughout time (measures of the 54 panels were taken every week for a total of 106 weeks). (B) Climate graph of Valencia city, displaying the mean annual temperatures and rainfall values (data source: AVAMET MX). (C) Solar panel voltage is shown and compared to Shannon diversity values at genus level of the detected 16S (grey line) and ITS (pink line) sequences. Seasons in which each sampling was performed are indicated in grey (winter), green (spring), pink (summer) and blue (autumn).

Solar panel efficiency, originally of roughly 20 Volts (V), displayed significant fluctuations in time and decreased during the first months of the experiment, but then recovered, and exhibited a very similar pattern during the next year (Fig. 1A). The efficiency was lower in the spring/summer months (between April and September), and this pattern was detected in both annuities, coinciding with the temperature increase and rainfall decrease recorded in Valencia, Spain (Fig. 1B). Bacterial diversity (Fig. 1C) and richness (Fig. S1A) increased during these spring/summer months and decreased during the autumn/winter period. In the case of fungi, the opposite pattern was observed: both the diversity (Fig. 1D) and the richness (Fig. S1B) decreased during the spring/summer months and increased during the autumn/winter period. Furthermore, seasonal decreases in bacterial richness and diversity (Fig. S2A) coincided with an increase in chloroplast sequences (Fig. S2B).

The mean relative abundance for each genus in time was calculated and the 15 most abundant bacteria and fungi were selected for further analysis (Table 1). The most abundant bacterial genera were *Modestobacter* (2.72%)*, Deinococcus* (2.52%)*, Sphingomonas* (2.44%), *Hymenobacter* (2.38%) and *Rubellimicrobium* (2.29%). On the other hand, the most abundant fungal genus was, by far, *Alternaria*, with 55.4% of mean relative abundance, followed by an unidentified fungi (5.6%) and an unidentified *Pleosporales* (5.4%) and by 13 other taxa that displayed between 0.5 and 2.5% of mean relative abundance.

**Table 1:** Fifteen bacterial and fungal genera with the highest mean relative abundance (MRA) throughout time obtained through 16S rRNA and ITS gene sequencing respectively.

Bacteria		Fungi	
Genus	MRA (%)	Genus	MRA (%)
*Modestobacter*	2.72	*Alternaria*	55.45
*Deinococcus*	2.52	*unidentified*	13.41
*Sphingomonas*	2.44	*Stemphylium*	2.56
*Hymenobacter*	2.38	*Cladosporium*	1.96
*Rubellimicrobium*	2.29	*Neocatenulostroma*	1.60
*Methylobacterium*	2.15	*Aureobasidium*	1.56
*Lactobacillus*	1.62	*Filobasidium*	1.49
*Skermanella*	1.41	*Coniosporium*	1.44
*Roseomonas*	1.29	*Nigrospora*	1.29
*Geodermatophilus*	1.15	*Knufia*	1.26
*Arthrobacter*	1.14	*Phaeosphaeria*	0.75
*Blastococcus*	1.09	*Sporobolomyces*	0.58
*Bacillus*	1.39	*Vishniacozyma*	0.55
*Microbispora*	1.12	*Symmetrospora*	0.54
*Paracoccus*	0.95	*Trebouxia*	0.51

**Fig. 2:**
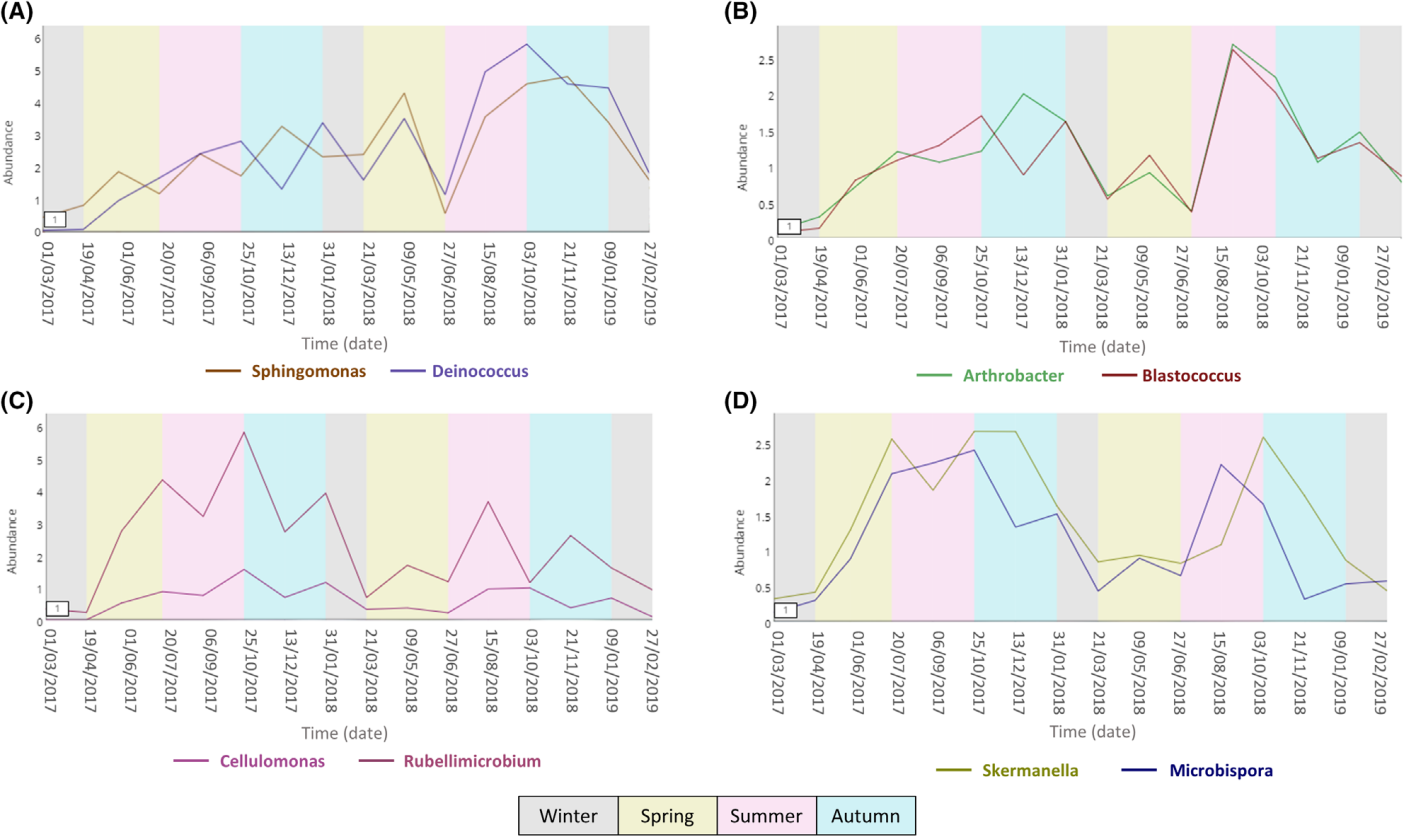
Trend plots of taxa that display a similar behaviour over time. A. *Sphingomonas* and *Deinococcus*. B. *Arthrobacter* and *Blastococcus*. C. *Cellulomonas* and *Rubellimicrobium*. D. *Skermanella* and *Microbispora*. These taxa were identified with TIME using a dynamic time warping (DTW) algorithm (Baksi *et al*., 2018). Seasons in which each sampling was performed are indicated in grey (winter), green (spring), pink (summer) and blue (autumn).

Fluctuations throughout time were observed for the 15 most abundant bacterial and fungal taxa (Fig. S3). A close‐up look at the most abundant taxa during the first 21 weeks (Fig. S4), revealed that *Lactobacillus*, *Bacillus, Sphingomonas* and *Hymenobacter* are among the first to arrive, and that the abundance of *Sphingomonas* increases during the first 14 weeks, remaining more or less stable after that. On the other hand, on weeks 14 and 21, there is a general increase in abundance of the most abundant taxa, although this increase is especially pronounced for *Rubellimicrobium, Modestobacter, Skermanella* and *Microbispora*, whereas other taxa, such as *Sphingomonas*, *Hymenobacter* or *Deinococcus* remain constant. Interestingly, several of the most abundant bacteria displayed similar temporal profiles: *Sphingomonas* and *Deinococcus* (Fig. 2A), *Arthrobacter* and *Blastococcus* (Fig. 2B), *Cellulomonas* and *Rubellimicrobium* (Fig. 2C), and *Skermanella* and *Microbispora* (Fig. 2D).

**Fig. 3:**
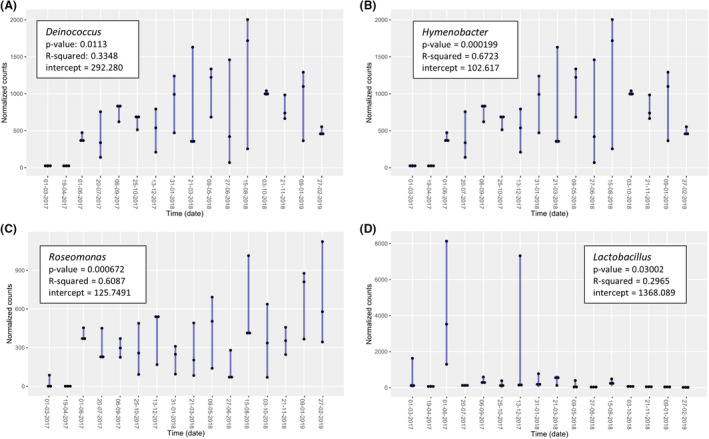
Statistically significant positive (A,B,C) and negative (D) trends observed in bacterial genera throughout time and calculated using Prais–Winsten estimation (*P*‐value < 0.05) Reported *P*‐values were calculated by applying the normalization of EdgeR package. R‐squared and intercept values are also indicated. The black dots indicate the normalized abundance for each of the three replicates.

**Fig. 4:**
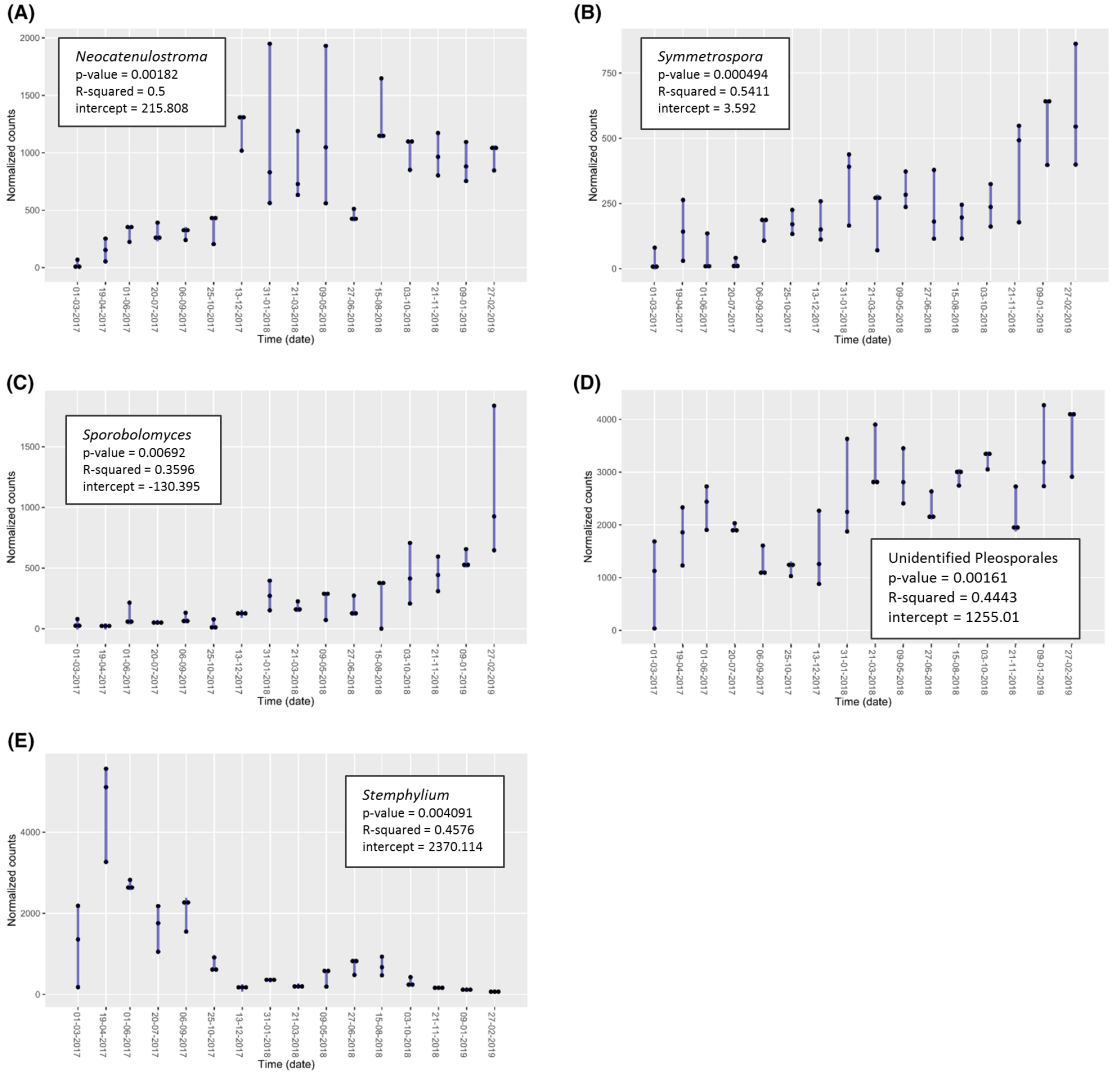
Statistically significant positive (A,B,C,D) and negative (E) trends observed in fungal genera throughout time and calculated using Prais–Winsten estimation (*P*‐value < 0.05). Reported *P*‐values were calculated by applying the normalization of EdgeR package. R‐squared and intercept values are also indicated. The black dots indicate the normalized abundance for each of the three replicates.

Despite the fluctuations observed, only several bacterial and fungal taxa displayed statistically significant increases or decreases throughout time (Figs 3 and 4). Specifically, *Deinococcus, Hymenobacter* and *Roseomonas* increased with time, whereas *Lactobacillus* decreased (Prais‐Winsten, *P*‐value < 0.05) (Fig. 3). Regarding fungi*, Neocatenulostroma, Symmetrospora, Sporobolomyces* and *Comoclathris* increased throughout time, whereas *Stemphylium* decreased (Fig. 4) (Prais–Winsten, *P*‐value < 0.05).

**Fig. 5:**
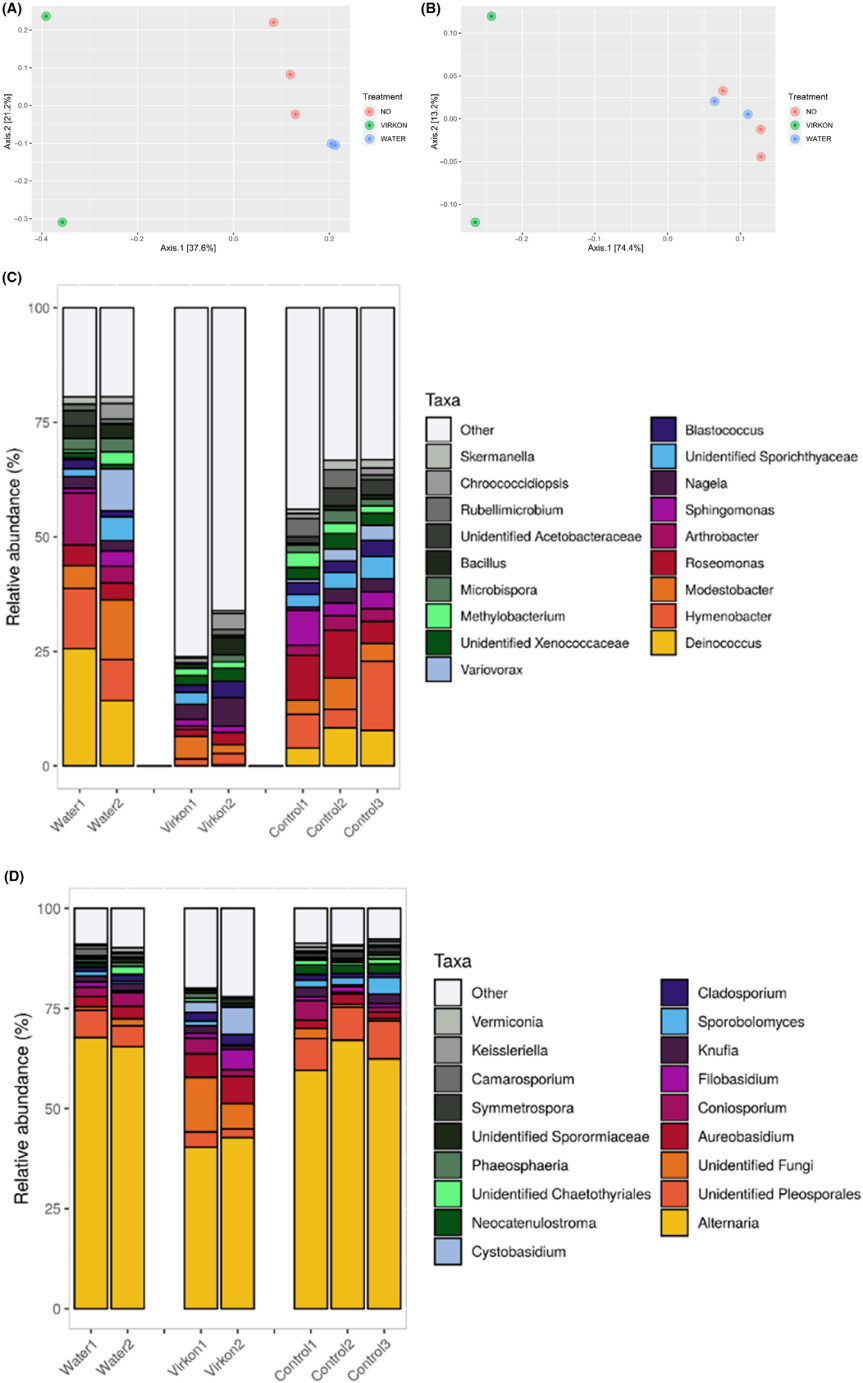
PCoA (using Bray–Curtis dissimilarities and full data) showing the variations in bacterial (A) and fungal (B) communities on solar panel surfaces as a result of not washing the surfaces in a period of 24 months, or washing them with water/Virkon every seven weeks. Taxonomic analysis of the bacterial (C) and fungal (D) communities in the three different conditions (surfaces unwashed for 2 years or washed with Virkon/water).

The effect of using a disinfectant on the microbial composition was studied using Rely + On Virkon (DuPont, Michigan, USA), a disinfectant that is routinely used to disinfect hard surfaces. This choice of disinfectant was based on the fact that Virkon does not generate fumes or strong odours, it is compatible with most hard non‐porous surfaces, it cleans and disinfects in one step, it has a long shelf life (2 years for the tablet format) and it is effective as determined by European EN standards (bactericidal, fungicidal and virucidal efficacy). Furthermore, in a 1% solution it is non‐irritating to eyes and skin. Solar panels that were cleaned with Virkon displayed very different bacterial profiles (Fig. 5A) when compared with the two types of controls (either dipped in sterile water or untreated, both of which displayed a more distant profile in comparison with the Virkon‐treated solar panels). Specifically, the panels treated with Virkon were characterized by the almost complete disappearance of *Deinococcus*, and by the increase of ‘other’ taxa, which corresponded mainly to the phyla *Proteobacteria, Bacteroidetes, Firmicutes* and *Actinobacteria* (Fig. 5C). On the other hand, differences were also observed in the fungal communities of the Virkon‐treated panels in comparison with the control treatments (Fig. 5B). Specifically, Virkon‐treated surfaces displayed a decrease in general diversity, an increase in the relative abundance of *Cystobasidium* and *Filobasidium,* as well as a slight increase in the abundance of taxa assigned to ‘other’, which corresponded mainly to the phyla *Pleosporales, Dothideales, Capnodiales and Tremellales* (Fig. 5D). It is important to note that the PCoA plots did not change substantially when only the most abundant 15 genera were used (data not shown). Regarding the effect on efficiency of cleaning the solar panels with water or Virkon, in general the produced voltage increased after cleaning, independently of the method used (Fig. S5).

## Discussion

Our results reveal that the microbial communities inhabiting solar panel surfaces change in time and experience seasonal variations. The microbial composition is characterized by a set of highly resistant bacterial genera (*Deinococcus, Hymenobacter, Roseomonas*) and fungi (*Alternaria*, among others), which are marginally present on the panels at the beginning of the experiment, but increase in frequency and become dominant by the end of the experiment. Some of the most abundant bacterial genera, such as *Hymenobacter, Modestobacter* and *Deinococcus*, have in fact previously been isolated from warm, irradiated environments, such as arid soil crusts or hyper‐arid desert soils (Reddy and Garcia‐Pichel, 2013; Busarakam *et al*., 2016; Gundlapally and Garcia‐Pichel, 2017), and they have also been reported as frequent taxa inhabiting solar panel surfaces (Dorado‐Morales *et al*., 2016; Tanner *et al*., 2018; Porcar *et al*., 2018). In fact, the microbial communities inhabiting solar panel surfaces around the world are similar in both functional and phylogenetic terms (Tanner *et al*., 2018; Porcar *et al*., 2018), suggesting the presence of not only common strong selective pressures (leading to functional similarity), but also of common structuring principles (leading to phylogenetic conservation) that include, among others, assembly history (the timing and order in which species arrive) and priority effects (the imprint of arrival order on community structure) (Carlström *et al*., 2019). Interestingly, *Deinococcus* and *Hymenobacter* have been proposed as biomarkers for desert airborne bacteria (Meola *et al*., 2015), indicating that a possible source of the solar panel microbiome could be the airborne transport of dust particles from deserts.

The most abundant bacterial taxa detected in this work (mean value throughout time) are consistent with those previously described to inhabit solar panel surfaces (Dorado‐Morales *et al*., 2016; Tanner *et al*., 2018; Porcar *et al*., 2018) and other radiation‐exposed environments, suggesting that the strong selection pressure imposed by solar radiation and other factors, such as desiccation, temperature or limited nutrient availability, is what shapes the microbial communities in these environments. For example, a previous study reported that concrete walls exposed to sunlight and ionizing radiation in Chernobyl proved to harbour similar communities to those present in a sun‐exposed environment from a control area (without ionizing radiation), and these were dominated by *Actinobacteria*, *Deinococcales* and pigmented ascomycete fungi (Ragon *et al*., 2011). Similar communities, dominated by *Actinobacteria, Cyanobacteria, Proteobacteria* and *Deinococcus‐Thermus*, have also been detected on other stone surfaces around the world, including Roman stone ruins in North Africa (Louati *et al*., 2019) and historic Scottish monuments (Suihko *et al*., 2007).

During the first weeks of colonization, members of the genus *Sphingomonas* were among the first taxa whose abundance increased on solar panel surfaces, suggesting a crucial role of this taxa in the establishment of the subaerial biofilm. This is not the first time that *Sphingomonas* spp. has been described to initiate biofilm formation (Bereschenko *et al*., 2010), and its contribution to biofilm formation is largely associated to its ability to secrete exopolysaccharides (EPS) (Venugopalan *et al*., 2005). At a larger time‐scale (24‐months), the most abundant taxa detected on solar panel surfaces were *Modestobacter, Deinococcus, Sphingomonas, Hymenobacter, Rubellimicrobium* and *Methylobacterium,* several of which (*Deinococcus*, *Hymenobacter* and *Roseomonas*) displayed an increase in abundance throughout time. These genera are known to contain radiation‐resistant (Su *et al*., 2014; Lee *et al*., 2017; Kim *et al*., 2018; Lim *et al*., 2019) and biofilm‐forming (Kolari *et al*., 2002; Saarimaa *et al*., 2006; Simões *et al*., 2010) species, traits that could contribute to their success in this environment. In the case of *Methylobacterium* species, these have shown the ability to form biofilms, adhere to polystyrene surfaces and tolerate desiccation and low nutrient conditions (Kolari *et al*., 2002; Simões *et al*., 2010; Yano *et al*., 2013). On the other hand, *Deinococcus* has been found to adhere to paper surfaces in industrial environments, acting as an intermediate for the adhesion of other bacteria (Kolari *et al*., 2002; Saarimaa *et al*., 2006). Thus, *Deinococcus* may play a role in both establishing and intermediating in the biofilm formation on solar panels. Furthermore, previous glass‐adhesion experiments with strains isolated from solar panel surfaces revealed that species belonging to the genus *Arthrobacter*, *Methylobacterium*, *Deinococcus* and *Hymenobacter* displayed a high ability to colonize glass surfaces (Dorado‐Morales *et al*., 2016).

The increase in abundance of several marker taxa is linked to the hypothesis that, after inoculation on the surface (i.e., via wind carrying desert soil, as suggested by the presence of *Deinococcus* and *Hymenobacter*), some of these taxa, namely those able to resist the extreme conditions inherent to solar panel surfaces, begin to form biofilm structures. In fact, high temperatures and poor nutrient conditions, as the ones that characterize solar panel surfaces, have been described to enhance biofilm formation (Yin *et al*., 2019), and these biofilms could in turn protect the microbial community from other environmental stressors. For example, in *Deinococcus geothermalis,* biofilm formation has been linked to an increased desiccation resistance, although it has also been linked to a decrease in UV resistance due to the photodissociation of water molecules retained in the EPS matrix, leading to increased ROS concentrations (Frösler *et al*., 2017). On the other hand, biofilm structures have also been described to protect against UV‐radiation due to physical shading (Yin *et al*., 2019). Interestingly, several bacterial taxa displayed very similar profiles throughout time, suggesting an interdependence between these genera. Whether this dependence is nutritional (i.e., auxotrophic complementation), physical (protection through biofilm formation) or due to another cause remains unknown. A recent study by Carlström *et al*. (2019) on the assembly rules of phyllosphere microbiota revealed that, once established, an initial microbial community is relatively robust and difficult to perturb through the introduction of new species. Nevertheless, in this previous study, single‐strain drop out experiments revealed the importance of key taxa in shaping community structures, mainly by affecting (either positively or negatively) strains with low abundance. In this sense, the initial weeks of colonization of solar panel surfaces are critical for the establishment of the final community, and the perturbation of certain strains due to seasonal/environmental variations could lead to the similar profiles observed for several bacterial taxa throughout time. In fact, Carlström *et al*. (2019) described predominantly (around 75%) inhibitory interactions among strains, although one of the two strains displaying positive interactions was found to be *Arthrobacter,* which we also detected in our experimental conditions, displaying a similar behaviour to *Blastococcus* (possibly due to a positive interaction).

In general, bacteria dominated the surface of the panels during the spring/summer period, whereas fungi were more abundant in autumn and winter, very likely linked to the moisture levels during the typically rainy autumn period and the relatively cool Mediterranean winter. Soiling has been reported to increase during low rainfall periods which, as well as affecting the performance of photovoltaic systems (Kimber *et al*., 2006), could also act as a nutrient source, leading to a larger accumulation of bacteria on the surfaces. On the other hand, fungi displayed an increase in richness and diversity in the autumn/winter period, which is consistent with several previous studies. For example, members of the genera *Alternaria, Cladosporium* and *Stemphylium,* among others, display increased ambient concentrations during high relative humidity periods (Llorente *et al*., 2012; Priyamvada *et al*., 2017). Furthermore, it has been shown that filamentous fungi can form biofilms when they grow on surfaces (Harding *et al*., 2009). Indeed, fungi are great candidates to live on surfaces as they secrete extracellular enzymes, they have an absorptive nutrition mode and they can easily invade surfaces due to the apical hyphal growth (Wessels, 1993). The most abundant taxa belonged to the genus *Alternaria*, consistent with the observation by Shirakawa *et al*. (2015), in which melanized *Ascomycetes* dominated the subaerial biofilms located on solar panel surfaces. The abundance of *Alternaria* on solar panel surfaces and other subaerial biofilms could be explained by the abundance within the species belonging to this genus of pathways for melanin biosynthesis, a pigment that confers protection against UV radiation and other environmental stressors (Kawamura *et al*., 1999; Tseng *et al*., 2011). Interestingly, some bacterial colonizers displayed significant tendencies to decrease throughout time. For example, the genus *Lactobacillus*, not known to be radiation resistant, generally associated to the human microbiota and characterized by including facultative anaerobic or microaerophilic bacteria, tended to decrease during the 2‐year experiment.

In our experimental conditions, seasonal fluctuations of solar panel efficiency (open circuit voltage) were observed, which we hypothesize are associated mainly to climatic conditions (specifically, reduced efficiency due to high temperatures, as previously reported) (Skoplaki and Palyvos, 2009; Omubo‐Pepple *et al*., 2009) and, to a lesser extent, to soiling and/or biofilm formation (a slight increase in efficiency was observed after rinsing the solar panels periodically with either water or Virkon). Nevertheless, although the use of water or Virkon yielded a similar increase in efficiency, the microbial community after each of those treatment was different. Specifically, the surfaces treated with water displayed a similar microbial composition than the untreated plates, whereas the ones treated with Virkon suffered from changes such as a clear decrease of the genus *Deinococcus,* which was not detected after cleaning the surfaces with Virkon. On the other hand, the fact that the water‐treated surfaces were similar, in taxonomic terms, to the untreated surfaces could provide an explanation regarding the stability throughout time of the solar panel microbiome: although rainfall (cleaning with water being a proxy of this) reduces soiling, is not enough to disrupt the microbial community inhabiting solar panel surfaces. Our results thus indicate that chemical agents can strongly modify the microbial composition of the panels, but do not seem to have an important effect on electric production, which is largely dependent on non‐biological factors such as dust accumulation and temperature fluctuations.

Taking into account these results, we hypothesize that solar panel surfaces are colonized by microorganisms that arrive through the deposition of soil and dust particles transported via wind. Then, in a very short time period, the microorganisms able to resist radiation and desiccation are selected by the environment and form robust biofilm structures. These biofilms then support the accumulation of other, lesser‐abundant organisms, leading to a stable community that is not altered by rainfall and, therefore, is robust throughout time.

This is the first work specifically designed to study, at a large scale and throughout a 2‐year time period, the colonization process of solar panel surfaces, focusing on both the fungal and bacterial communities. The most abundant bacterial genera detected (*Modestobacter, Deinococcus, Sphingomonas, Hymenobacter* and *Rubellimicrobium*) and the most abundant fungal genera (*Alternaria,* among others) are consistent with previous studies on solar panel microbiomes. Our results allow us to conclude that the presence of such taxa on solar panels is not the result of their mere accumulation from the surrounding environment, but corresponds to the final step of an ecological succession, in the frame of which extremophilic taxa adapted to the harsh conditions of solar panels are selected. Indeed, a significant increase of solar panel‐adapted genera such as *Deinococcus*, *Hymenobacter*, *Roseomonas* and *Neocatenulostroma)* as well as the decrease of non‐resistant, ubiquitous taxa (*Lactobacillus* or *Stemphyllium*) was recorded throughout the experiment. Nevertheless, this accumulation of microorganisms is not linked to a significant reduction in photovoltaic efficiency, which exhibits a seasonal variation and that is not improved by antiseptic compounds. It can be concluded that the microbial community is clearly modified by such compounds but that this fact is not linked to a clear benefit in terms of enhanced electric efficiency, at least under the Mediterranean conditions of our study.

## Experimental procedures

### Small‐scale solar farm construction

**Fig. 6:**
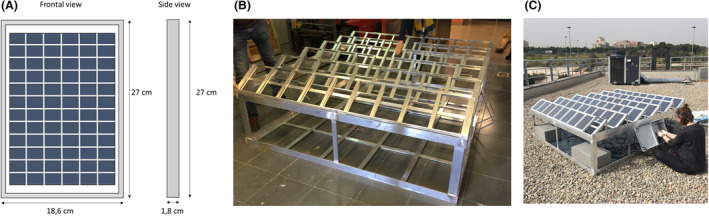
Experimental set‐up: 54 small‐sized solar panels (A) were set up on an aluminum chassis (B) and placed on the rooftop of a building in the Scientific Park of the University of Valencia in Paterna, Spain (C).

For this work, a small‐scale solar farm was built using 54 small‐sized solar panels (SOLARPOWER 5W‐12V, Xunzel Soluciones S.L., Mendaro‐Guipuzcoa, Spain) mounted on a aluminium frame designed *ad hoc* by the ICMUV Institute (Valencia, Spain; Fig. 6A). The surfaces of the panels were sterilized on‐site by cleaning them with 70% ethanol. Then, they were placed in the metallic structure (Fig. 6B), which had previously been placed on the roof of one of the buildings belonging to the Scientific Park of the University of Valencia (39°30′56.0″N 0°25′28.4″W) in an equator‐facing position (Fig. 6C). Furthermore, all the solar panels were electrically connected to two connection boxes placed at either side of the structure and that were sealed in order to avoid the entrance of water or environmental particles. Once a week (except on cloudy days), and for a period of two years, the efficiency of each solar panel was measured twice and both values were recorded.

### Solar panel sampling

Throughout the two‐year time period, the surfaces of four of the solar panels were subjected to a treatment with either a disinfectant or water, with the goal of comparing, at the end of the experiment, the microbial taxonomy of both groups. Every seven weeks, two solar panel surfaces were soaked in sterile distilled water for 10 min, and another two were soaked in a solution of Rely + On Virkon disinfectant at 10 g l^−1^, the working concentration recommended by the manufacturers (DuPont, Michigan, USA) for 10 min, followed by a rinse with sterile distilled water. After cleaning, these solar panels were left to dry in the sun for 10 min and then placed again in the metal structure. At the end of the 2‐year period, these four solar panels were sampled together with the final three (uncleaned during 2 years).

Additionally, every seven weeks, three solar panels were randomly selected and sampled. The selected solar panels were removed from the metallic frame, placed in sterile bags and transported to the laboratory. Then, the panels were placed in a laminar flow hood and the surfaces were washed with sterile phosphate‐buffered saline (PBS) using a sterile window cleaner. The resulting liquid was concentrated into a pellet by centrifugation, and all pellets were frozen at −20°C until required.

### DNA extraction, sequencing and bioinformatic analysis

All DNA extractions were performed using the Power Soil DNA isolation kit (MO BIO Laboratories, Carlsbad, CA, USA), and the resulting DNA was quantified using the QUBIT dsDNA HS‐high sensitivity kit (Invitrogen, CA, USA). NextSeq Illumina libraries were constructed, targeting the hypervariable V3 and V4 regions of the 16S gene (Forward = 5′ TCGTCGGCAGCGTCAGATGTGTATAAGAGACAGCCTACGGGNGGCWGCAG; Reverse = 5′ GTCTCGTGGGCTCGGAGATGTGTATAAGAGACAGGACTACHVGGGTATCTAATCC) and targeting the ITS region (Forward = 5′CTTGGTCATTTAGAGGAAGTAA3′; Reverse = 5′GCTGCGTTCTTCATCGATGC3′). Then, Illumina sequencing adaptors and dual‐index barcodes (Nextera XT index kit v2, FC‐131‐2001) were added, and libraries were normalized and pooled. The pools were loaded onto the MiSeq reagent cartridge v3 (MS‐102‐3003), spiked with 10% PhiX control and sequencing was conducted using paired‐ends on an Illumina MiSeq sequencing system. Rarefaction curves were saturated for all samples, indicating that sequencing was deep enough to assess all microbial diversity (Fig. S6). Mean values of 36 533 and 52 192 sequences were obtained for the 16S gene and the ITS region, respectively, with a minimum of 9669 and a maximum of 61 764 sequences for the 16S gene, and a minimum of 25 640 and a maximum of 68 942 sequences for the ITS region.

Raw Illumina sequences were analysed using Qiime2 (Boylen *et al*., 2019). Briefly, the quality of the reads was assessed with the Demux plugin, and the sequences subsequently corrected and trimmed via dada2. The taxonomy of each sequence variant was assigned employing the classify‐Sklearn module from the feature‐classifier plugin. greengenes (v. 13.8.99) and uniite (v. 7_99_01.12.2017) were used as reference databases for 16S rRNA and ITS taxonomic assignment respectively. For the time‐series analysis, taxonomy was collapsed into the genus level. For each sampling time and genus, an average of the three replicates sequence count was calculated. The web application TIME (Temporal Insights into Microbial Ecology) was used to analyse and represent the temporal distributions of the taxonomic profiles (Baksi *et al*., 2018), dividing the time period in four seasons: spring (21 March to 20 June), summer (21 June to 20 September), autumn (21 September to 20 December) and winter (21 December to 20 March).

The 15 most abundant genera were selected in order to study their temporary trends. Average sequence counts were calculated for each sampling time, and Prais–Winsten estimation was carried out for each genus using the 'Prais' R package. This linear model was applied for its ability to handle autocorrelation, which is usually found in time‐series data. Regressions were calculated using three approaches: with the raw abundance data, normalizing the data through rarefaction with respect to the sample with the lowest sequencing depth and applying the normalization of EdgeR package. All three approaches yielded the same result, and the *P*‐values indicated in Figures 3 and 4 were calculated with the edgeR approach. In all the statistically significant tendencies observed for bacteria and fungi, independently of the approach used, the *P*‐value was below 0.05.

## Conflict of interest

The authors declare no conflict of interest.

## Supporting information


**Fig. S1.** Solar panel efficiency measurements (blue dots) are shown and compared to the Richness at genus level of the detected 16S (grey dots) and ITS (pink dots) sequences (these measurements correspond to days in which samples were taken from the surface for genomic analysis). Seasons in which each sampling was performed are indicated in grey (winter), green (spring), pink (summer) and blue (autumn).

**Fig. S2.** (A) *Y*‐axes indicates bacterial Richness (green) and Shannon diversity index (purple) at genus level throughout time. (B) Taxonomic distribution of bacteria in time at class level. Seasons in which each sampling was performed are indicated in grey (winter), green (spring), pink (summer) and blue (autumn).

**Fig. S3.** Variation in % of abundance throughout time of the 15 bacterial (A) and fungal (B) genera with highest mean abundance. Graphs are separated for 5 genera at a time to facilitate visualization of the data and are ordered from more abundant (top) to less abundant (bottom). Seasons in which each sampling was performed are indicated in grey (winter), green (spring), pink (summer) and blue (autumn).

**Fig. S4.** Close up of the most abundant genera in the first 21 weeks of sampling.

**Fig. S5.** Change in open voltage (% of increase or decrease) after cleaning with Virkon or water. Values are shown for the two replicates of each condition (blue dots for plates treated with Virkon and orange dots for plates treated with water).

**Fig. S6.** Rarefaction curves for sequences corresponding to the 16S gene (A) and ITS region (B).
