# Supplementary figures and images for: Extremophilic microbial communities on photovoltaic panel surfaces: a two‐year study
Source: Microb Biotechnol. 2020 Jul 1;13(6):1819–30. doi: 10.1111/1751-7915.13620 (PMC7533311; doi:10.1111/1751-7915.13620)

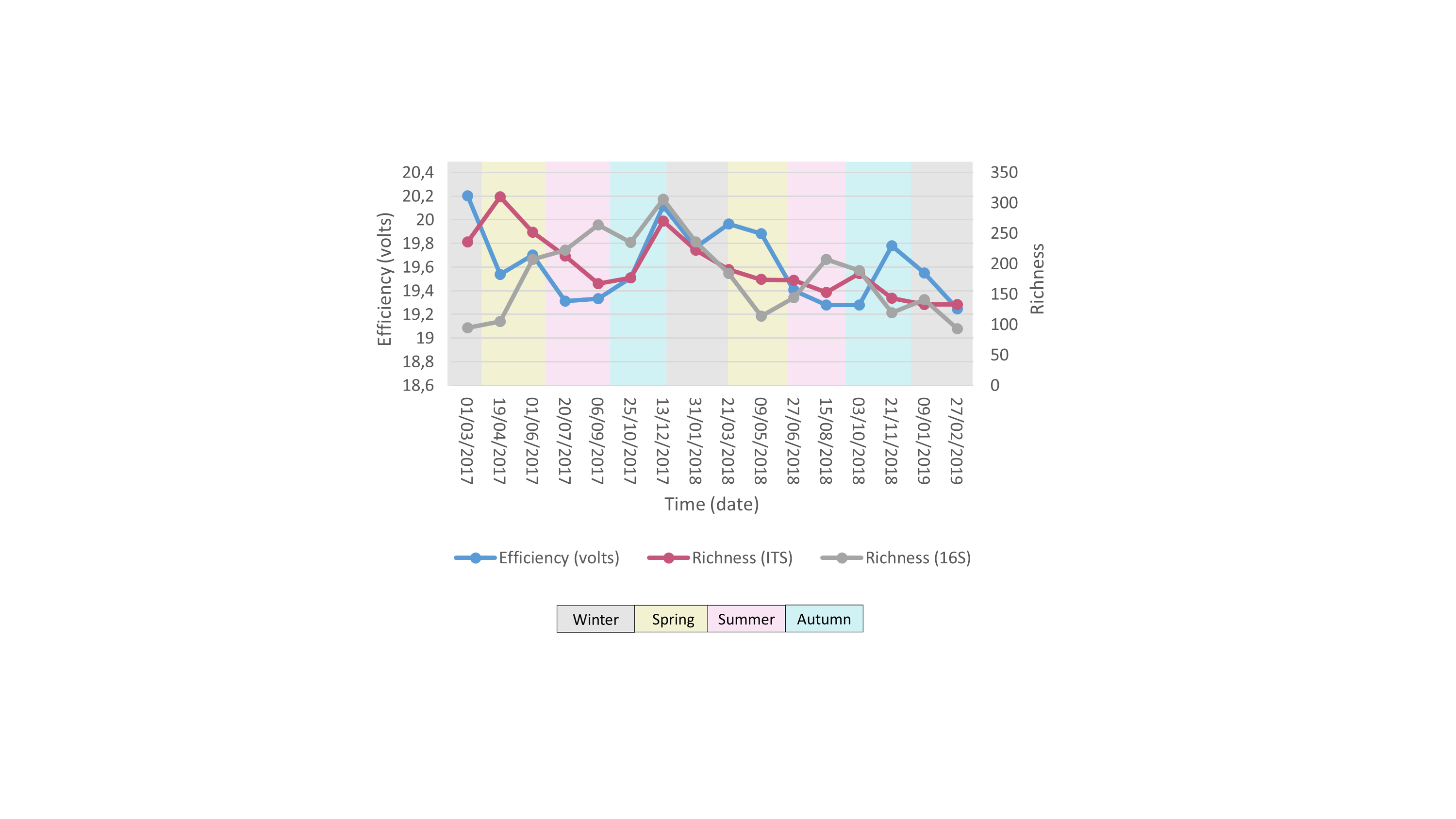

Supplement: Supplementary file 1 — Fig. S1. Solar panel efficiency measurements (blue dots) are shown and compared to the Richness at genus level of the detected 16S (grey dots) and ITS (pink dots) sequences (these measurements correspond to days in which samples were taken from the surface for genomic analysis). Seasons in which each sampling was performed are indicated in grey (winter), green (spring), pink (summer) and blue (autumn). [file MBT2-13-1819-s001.tif]

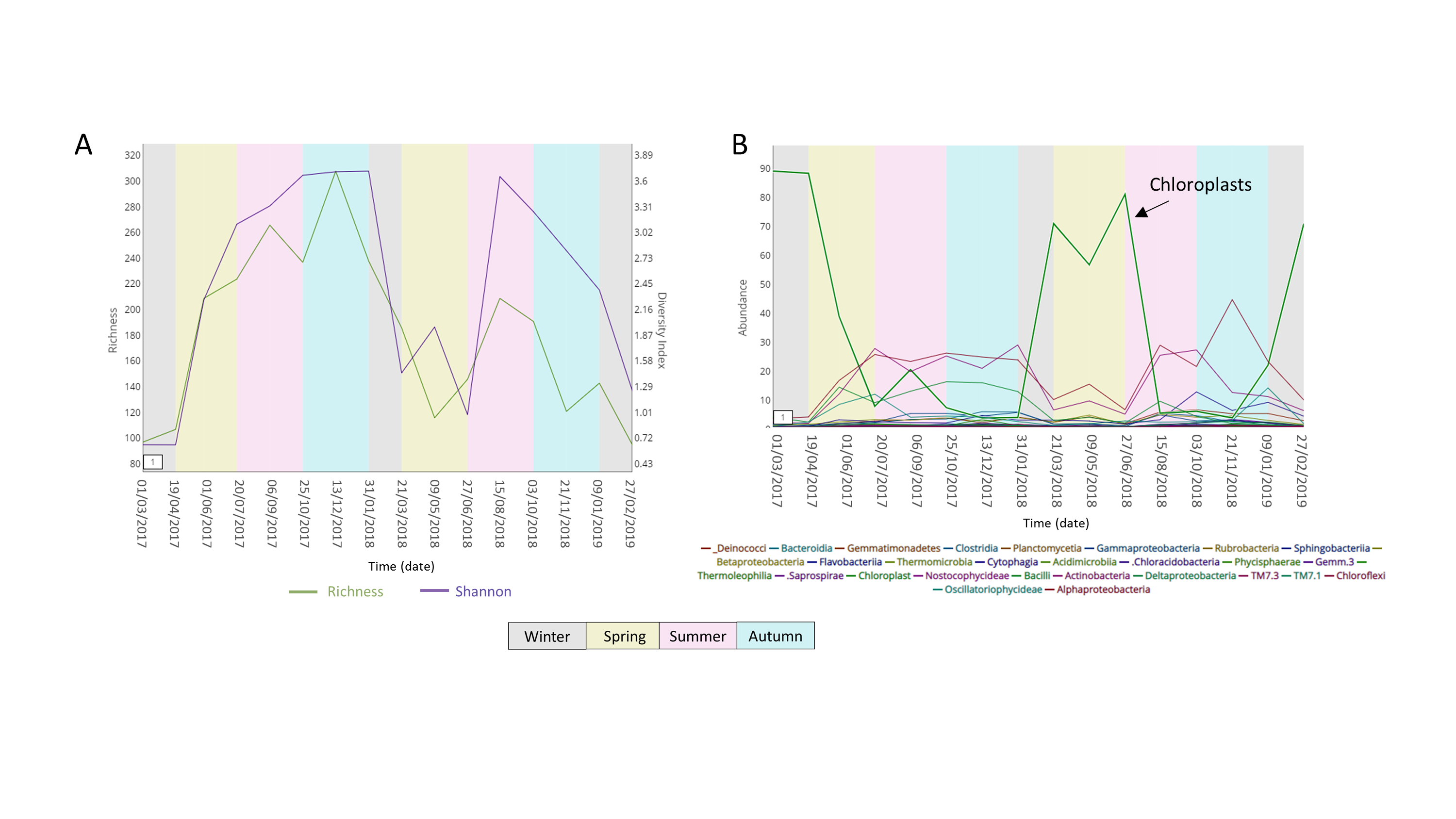

Supplement: Supplementary file 2 — Fig. S2. (A) Y‐axes indicates bacterial Richness (green) and Shannon diversity index (purple) at genus level throughout time. (B) Taxonomic distribution of bacteria in time at class level. Seasons in which each sampling was performed are indicated in grey (winter), green (spring), pink (summer) and blue (autumn). [file MBT2-13-1819-s002.tif]

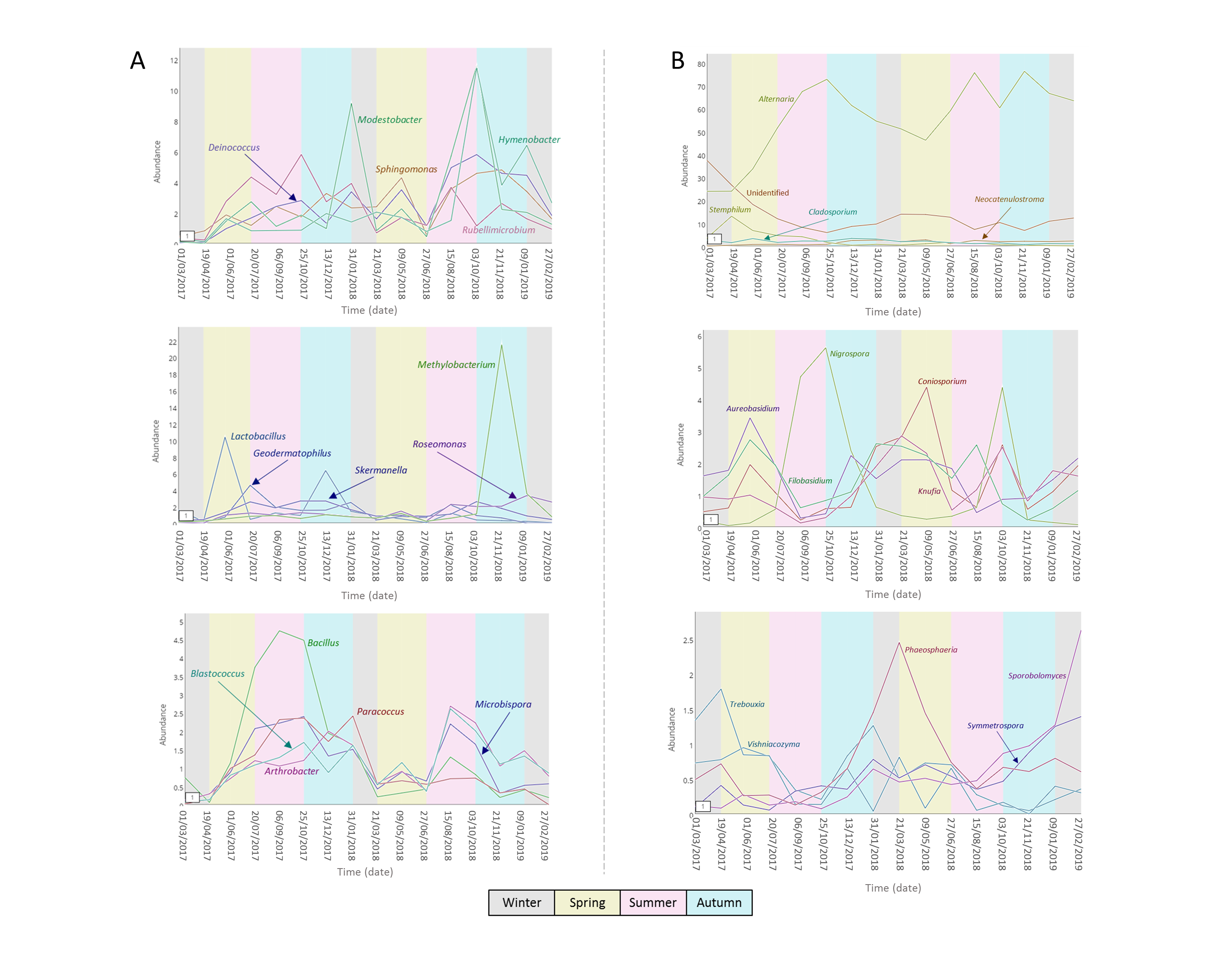

Supplement: Supplementary file 3 — Fig. S3. Variation in % of abundance throughout time of the 15 bacterial (A) and fungal (B) genera with highest mean abundance. Graphs are separated for 5 genera at a time to facilitate visualization of the data and are ordered from more abundant (top) to less abundant (bottom). Seasons in which each sampling was performed are indicated in grey (winter), green (spring), pink (summer) and blue (autumn). [file MBT2-13-1819-s003.tif]

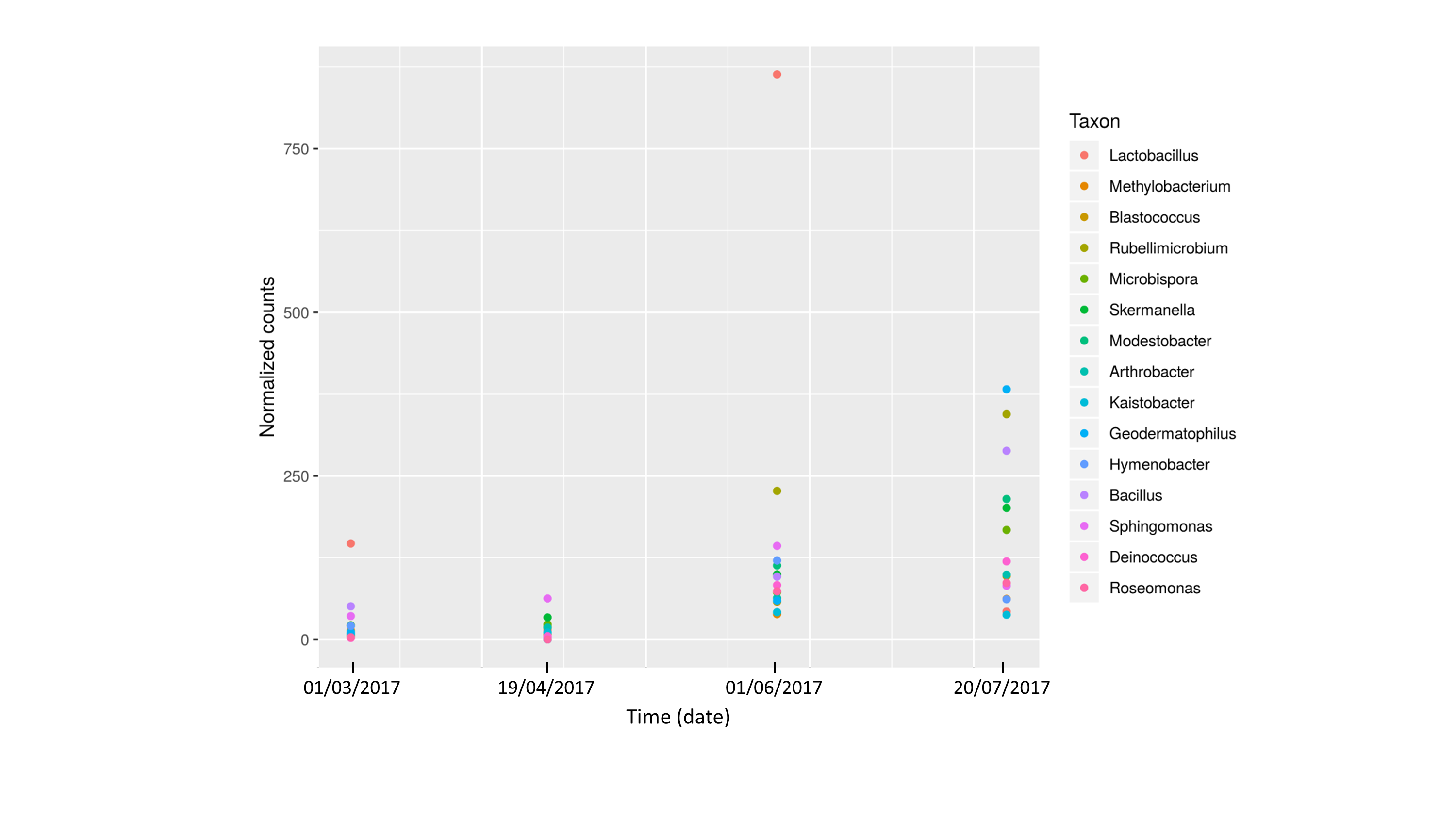

Supplement: Supplementary file 4 — Fig. S4. Close up of the most abundant genera in the first 21 weeks of sampling. [file MBT2-13-1819-s004.tif]

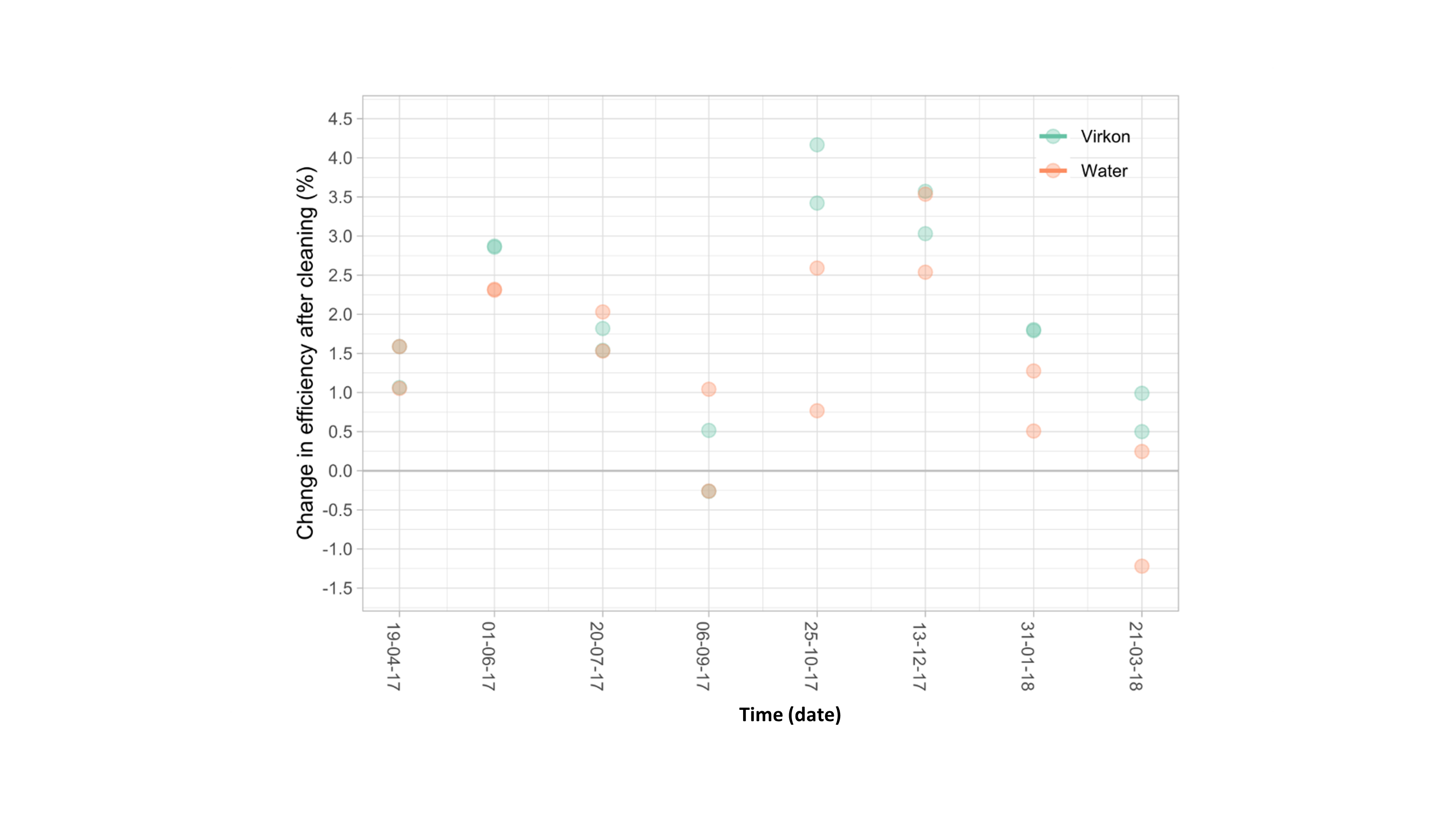

Supplement: Supplementary file 5 — Fig. S5. Change in open voltage (% of increase or decrease) after cleaning with Virkon or water. Values are shown for the two replicates of each condition (blue dots for plates treated with Virkon and orange dots for plates treated with water). [file MBT2-13-1819-s005.tif]

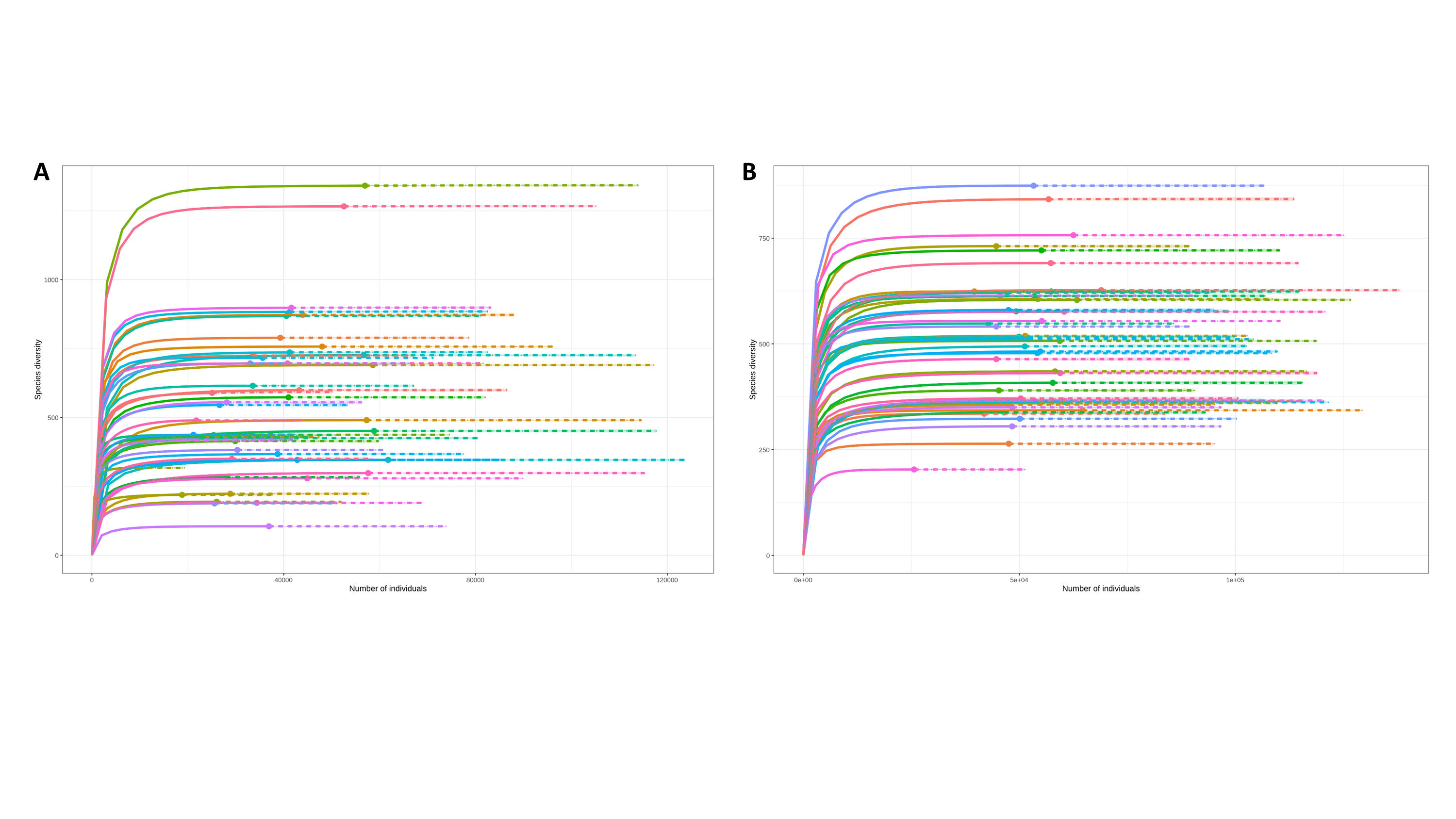

Supplement: Supplementary file 6 — Fig. S6. Rarefaction curves for sequences corresponding to the 16S gene (A) and ITS region (B). [file MBT2-13-1819-s006.tif]
